# Cybersickness in People with Multiple Sclerosis Exposed to Immersive Virtual Reality

**DOI:** 10.3390/bioengineering11020115

**Published:** 2024-01-24

**Authors:** Massimiliano Pau, Federico Arippa, Bruno Leban, Micaela Porta, Giulia Casu, Jessica Frau, Lorena Lorefice, Giancarlo Coghe, Eleonora Cocco

**Affiliations:** 1 Department of Mechanical, Chemical and Materials Engineering, University of Cagliari, 09123 Cagliari, Italy; arippaf@gmail.com (F.A.); bruno.leban@unica.it (B.L.); micaela.porta@unica.it (M.P.); giuliacasu.gc@outlook.it (G.C.); 2 Multiple Sclerosis Center, Binaghi Hospital, ASL Cagliari, 09123 Cagliari, Italy; jessicafrauneuro@gmail.com (J.F.); lorena.lorefice@hotmail.it (L.L.); gccoghe@gmail.com (G.C.); ecocco@unica.it (E.C.); 3 Department of Medical Sciences and Public Health, University of Cagliari, 09123 Cagliari, Italy

**Keywords:** virtual reality (VR), cybersickness, multiple sclerosis (MS), postural sway

## Abstract

Together with the wide range of possible benefits for the rehabilitation/training of people with multiple sclerosis (pwMS) and other neurologic conditions, exposure to immersive virtual reality (VR) has often been associated with unpleasant symptoms, such as transient dizziness, headache, nausea, disorientation and impaired postural control (i.e., cybersickness). Since these symptoms can significantly impact the safety and tolerability of the treatment, it appears important to correctly estimate their presence and magnitude. Given the existing data scarcity, this study aims to assess the existence and severity of possible adverse effects associated with exposure to immersive VR in a cohort of pwMS using both objective measurements of postural control effectiveness and subjective evaluations of perceived symptoms. To this aim, postural sway under upright quiet posture (in the presence and absence of visual input) of 56 pwMS with an Expanded Disability Status Scale score (EDSS) in the range of 0–6.5 (mean EDSS 2.3) and 33 unaffected individuals was measured before and after a 10-min immersive VR session and at 10 min follow-up on the basis of center of pressure (COP) trajectories. The severity of cybersickness symptoms associated with VR exposure was also self-rated by the participants using the Italian version of the Simulator Sickness Questionnaire (SSQ). Temporary impairments of postural control in terms of significantly increased sway area were observed after the VR session only in pwMS with mild–moderate disability (i.e., EDSS in the range of 2.5–6.5) in the presence of visual input. No changes were observed in pwMS with low disability (EDSS 0–2) and unaffected individuals. In contrast, when the visual input was removed, there was a decrease in sway area (pwMS with mild–moderate disability) and COP path length relating to the use of VR (pwMS with mild–moderate disability and unaffected individuals), thus suggesting a sort of “balance training effect”. Even in this case, the baseline values were restored at follow-up. All participants, regardless of their status, experienced significant post-VR side effects, especially in terms of blurred vision and nausea. Taken together, the findings of the present study suggest that a short immersive VR session negatively (eyes open) and positively (eyes closed) impacts the postural control of pwMS and causes significant disorientation. However, such effects are of limited duration. While it is reasonable to state that immersive VR is sufficiently safe and tolerable to not be contraindicated in the rehabilitation/training of pwMS, in order to reduce possible negative effects and maximize the efficacy, safety and comfort of the treatment, it appears necessary to develop specific guidelines that consider important factors like individual susceptibility, maximum exposure time according to the specific features of the simulation, posture to adopt and protocols to assess objective and perceived effects on participants.

## 1. Introduction

Although early pioneering attempts to create alternate reality scenarios with which humans could interact date back to the 1960s, the technology necessary to develop computer-controlled virtual reality (VR) systems was only made available a decade later [1] and, at least initially, it was specifically employed to reproduce complex simulation scenarios typical of the aerospace field with the purpose of serving as a training tool for military pilots and astronauts [2]. Instead, actual mass-consumer VR systems, mostly intended for entertainment purposes, were launched on the market 30 years ago, and those specifically designed for home-based use are even more recent.

Despite the initial trend, which indicates the prevalent use of VR for training and ludic purposes, researchers and practitioners involved in neurorehabilitation quickly realized the great potential of VR to improve the effectiveness of physical rehabilitation programs and possibly extend their effects by integrating home-based training by exploiting the low-cost and ease of use of commercial consoles (like Nintendo Wii, Kyoto, Japan, Microsoft Kinect, Redmond, WA, USA, etc.). As a result, VR has become a widespread technique that has been recognized as being beneficial for improving the ambulation, mobility, balance and upper limb function of individuals affected by a wide range of neurologic conditions (i.e., cerebral palsy, stroke, acquired brain injury, Parkinson’s disease, multiple sclerosis, etc.), despite the lack of specific guidelines on its use and optimal dosage, not to mention safety concerns; all these aspects, have not been adequately addressed to date [3].

In the last two decades, several studies have been carried out to verify the effectiveness of VR either as stand-alone approaches or add-ons to physical rehabilitation programs designed for people with multiple sclerosis (pwMS). Although they suffer from high heterogeneity in terms of employed devices, protocols, and outcome measures, the overall analysis of their results suggests that VR-based training can significantly improve fatigue symptoms, quality of life, gait, and the postural control of pwMS, with a magnitude of positive effects at least equal to, or greater than, conventional exercise [4,5]. Moreover, although gait and balance seem to be the most targeted functions for this kind of approach, there is preliminary evidence about the effectiveness of VR-based treatments in terms of upper limb (UL) motor function, dexterity, and hand grip strength [6]. In this context, recent studies have demonstrated that VR-only training is able to improve clinical measures of UL function [7] as well as performance of tasks associated with activities of daily living [8], and specific software tailored on the needs of pwMS starts to show up [9,10].

However, along with these interesting and promising features, the integration of VR into rehabilitative programs is not free from criticalities, particularly in the case of immersive VR. The contents of this particular type of VR are delivered through a head-mounted display (HMD), which provides the most realistic simulation experience by preventing users from receiving the sensory flow of information from the real world. Such configuration enhances a strong subjective state of “presence” in the simulated environment [11]. Although applications of immersive VR in neurorehabilitation are still quite limited, it has been hypothesized that individuals with neurological conditions who experience immersive VR scenarios would tend to act as they normally would do in real-life situations and, for this reason, are more likely to experience a successful transference of skills and knowledge acquired in VR to the real world [12,13]. However, along with such promising benefits, it is not uncommon to observe in those exposed to immersive VR (even for short times) unpleasant side effects typically expressed in the form of transient dizziness, headache, nausea, disorientation, and impaired postural control, which can indiscriminately affect people with neurological conditions and healthy individuals [3,14]. Such symptoms are usually pooled under the umbrella of “cybersickness” [15], a term coined to define a specific kind of motion sickness that occurs as a result of exposure to virtual/simulated environments. Cybersickness due to immersive VR is often assessed using questionnaires based on the self-reporting of symptom severity and, among them, the most widely employed [16] is the Simulator Sickness Questionnaire (SSQ) developed by Kennedy et al. in 1993 [17], which includes 16 symptoms (see Table 1 for details) grouped into three non-mutually exclusive domains (i.e., nausea, oculomotor disturbance, and disorientation). However, researchers also explored the possibility of objectively assessing cybersickness by analyzing changes occurring due to exposure to VR in postural sway, cardiac, brain, gastric or electrodermal activity [16].

To the best of our knowledge, only three studies specifically investigated cybersickness features in pwMS exposed to VR. In two of them [18,19], the SSQ was administered in combination with either analysis of galvanic skin response or electroencephalography to eight pwMS with the Expanded Disability Status Scale (EDSS, a method developed by John Kurtzke in 1983 to quantify disability in MS based on examination by a specialized neurologist, which is widely used in clinical routine [20]) scored in the range of 4–4.5 who underwent a simulated driving task. The authors found that cybersickness presented with different features in pwMS when compared to unaffected individuals, thus suggesting that specific techniques should be adopted to minimize the possible negative effects associated with VR administration within a rehabilitative/training program. Instead, the study of Ferdous et al. [21] investigated the effects associated with the use of a static rest frame to improve postural stability in seven pwMS (with EDSS score between 3 and 4.5) exposed to virtual and augmented reality. In this case, it was found that the inclusion of a static rest frame in the VR setup positively affected the balance of pwMS, thus improving their experience in the case of administration of VR using HMD [21].

Based on these considerations, the present study aims to expand knowledge about the possible adverse effects associated with exposure to immersive VR in pwMS as follows: (1) by testing a larger sample of pwMS, characterized by a wide range of EDSS scores, in such a way as to verify whether disability is a factor able to influence the magnitude and persistence of cybersickness and (2) by objectively characterizing the effect of exposure to VR in terms of an alteration in postural control effectiveness, assessed through postural sway measurement. Such data, in combination with the subjective perception of cybersickness, will be critically analyzed to provide some practical guidelines about the use of immersive VR in rehabilitative/training contexts.

## 2. Materials and Methods

### 2.1. Participants

During the period January–June 2023, a convenience sample of 56 pwMS (46 female and 10 males with a mean age of 45.3 years) with an EDSS score in the range of 0–6.5 (mean EDSS 2.3) were enrolled in the study at the Multiple Sclerosis Center of Cagliari (Binaghi Hospital, ASL Cagliari, Cagliari, Italy) after evaluation by a neurologist expert in MS (EC, GC, LL, JF). The main inclusion criteria were as follows: age > 18 years, having received a diagnosis of MS according to the 2017 McDonald criteria [22], being naïve to the use of immersive VR devices and being able to independently and safely maintain an upright posture for at least 60 s both in the presence and absence of visual input. The exclusion criteria were as follows: the existence of concurrent neurological, vestibular and orthopedic conditions that severely affect balance (based on clinical judgment), the existence of visual and hearing impairments that can prevent the correct perception of the visual and auditive stimuli generated by the VR device, and the presence of severe cognitive impairment. The participants were stratified into two groups according to their disability level as follows:

- Low disability (EDSS in the range 0–2, *n* = 34).
- Mild–moderate disability (EDSS in the range 2.5–6.5, *n* = 22).

A control group of unaffected individuals (*n* = 33, mean age 47.6 years) was composed through recruitment among the staff of the Binaghi Hospital and the University of Cagliari. The study was approved by the local Ethics Committee (protocol number 102/2018/CE) and conducted according to the principles expressed in the World Medical Association Declaration of Helsinki and its latest amendments. All participants signed an informed consent form. Their main anthropometric and clinical features are reported in Table 2. Of note, none of them had previous experience with immersive VR systems.

### 2.2. General Outline of the Study

**Figure 1:**
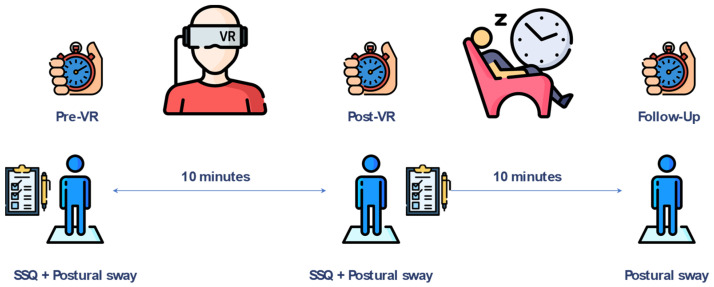
Outline of the study.

The design of the study is summarized in Figure 1. In short, we assessed the possible adverse effects concerning a 10 min exposure to a highly stimulating immersive VR simulation by collecting data on postural control effectiveness and perceived discomfort. The former aspect was explored by means of postural sway analysis (described in detail later); in this regard, the participants were tested immediately before and after the VR session and after 10 min of complete passive rest (follow-up). This time period was chosen according to previous studies, which was considered suitable to fully restore the baseline conditions [23,24,25]. Instead, the perceived discomfort was assessed only before and after the VR session using the SSQ questionnaire previously mentioned. Although in most studies on cybersickness, the SSQ is usually administered only after exposure to the simulated environment (implicitly assuming that no symptoms are present before the VR session), it has been recently recognized that most individuals are likely to approach a VR session with preexisting cybersickness symptoms [26], and thus the actual impact of VR exposure might be overestimated. In the case of pwMS, this aspect is even more critical as some of the symptoms included in the questionnaire to describe cybersickness (i.e., fatigue, dizziness, difficulty concentrating, etc.) overlap with those typical of MS. Thus, only the comparison of the SSQ scores calculated before and after the WR session can provide a reliable indicator of the actual perceived negative effects originating from its use.

### 2.3. The Immersive VR System

A commercially available low-cost VR headset (Meta Quest 2, formerly known as Oculus Quest 2) developed by Meta Platforms Inc. (Cambridge, MA, USA) and released in 2020 was employed for the present study. The main component of the Quest 2 is an HMD equipped with a singular, fast-switch LCD panel with a resolution of 1832 × 1920 pixels per eye and a refresh rate of up to 120 Hz. The device can accommodate interpupillary distances between 56 and 70 mm. Arm and hand movements, as well as other kinds of actions within the virtual scene, are managed by the user by means of two dedicated controllers. The software selected for the experimental tests was a rollercoaster simulation (“Epic Rollercoaster”, B4T Games, Brazil, http://b4t.games, accessed on 25 December 2023) previously employed in similar studies on cybersickness due to their ability to strongly stimulate visual input giving a very realistic illusion of movement. In this scenario, the player basically experiences a simulated view and motion associated with a ride on a rollercoaster train.

### 2.4. Postural Sway Measurement

Postural control system performance under upright posture conditions was assessed by means of postural sway measurements, which represent the most adopted instrumental technique to objectively assess VR-induced sickness [16]. This was carried out by processing the center of pressure (COP) time series acquired at a 50 Hz frequency using a pressure platform (FDM-S, Zebris Medical GmbH, Isny, Germany) composed of 2560 capacitive sensing elements arranged in a 64 × 40 matrix and connected via a USB interface to a personal computer. The participants were required to stand barefoot on the platform, having their feet positioned according to the recommendations of the International Society of Posturography (now the International Society of Posture and Gait Research) reported by Kapteyn et al. [27], that is, with the feet axis oriented approximately at 30° and an inter-malleolar distance of 8–10 cm. A reference mask with the drawing of two footprints properly positioned was placed on the top of the platform to ensure a common reference position. The participants were then invited to maintain a stable and relaxed position with arms freely positioned along their sides and gaze fixed on a target image placed at a distance of 3 m. Three 30 s trials were acquired in random order for two conditions, namely in the presence and absence of visual input. A suitable rest time between the trials was allowed at the participant’s request.

The raw COP trajectories were low-pass filtered (10 Hz cutoff; 4th-order Butterworth; bidirectional) and then post-processed with a custom-developed Matlab^®^ routine to calculate the following sway parameters:

- Sway area (mm^2^, 95% confidence ellipse);
- COP path length (mm, the overall length of the trajectory followed by the COP during the trial);
- COP maximum displacements in antero–posterior (AP) and medio–lateral (ML) direction (mm, the difference between the maximum and the minimum coordinate assumed by the COP during the trial);
- COP velocities in the antero–posterior (AP) and medio–lateral (ML) directions (mm/s).

The mean value of the three trials for eyes open and eyes closed conditions was calculated for each parameter and considered representative of a certain participant.

### 2.5. Subjective Rating of Cybersickness: The Simulator Sickness Questionnaire (SSQ)

As previously mentioned, the Italian version of the SSQ was used to assess the presence and severity of unpleasant symptoms associated with VR exposure. Originally proposed in 1993 [17] for military purposes, this tool is most commonly used to quantify the degree of sickness in users of modern VR systems [16]. Basically, the SSQ requires an individual to rate the severity of 16 symptoms on a 4-point scale (from 0 = no perception to 3 = severe perception). The symptoms are pooled into three non-mutually exclusive domains, namely nausea, oculomotor disturbance, and disorientation, whose score is calculated as the sum of the scores assigned to each symptom multiplied by a constant scaling factor. The total score of the SSQ is then obtained by combining those of the three sub-scales (for further details on score calculation and practical suggestions about the correct use of the SSQ, see Bimberg et al. [28]), and its interpretation is quite straightforward as higher values are indicative of stronger sickness symptoms, and thus the VR exposure is perceived as more disturbing. Although SSQ scores are influenced by various factors like the type of simulation, exposure time, sex, user’s habit to simulated environments, etc., reference values (for total score) in previous studies on individuals with neurologic conditions reported total score values following immersive VR exposure in the range of 17–33 [18,29,30].

### 2.6. Statistical Analyses

The existence of possible effects associated with VR exposure on postural sway and SSQ scores was investigated by means of a two-way analysis of variance for repeated measures (RM-ANOVA), considering the group (unaffected individuals; MS with low disability; and MS with mild–moderate disability) and time (pre-VR; post-VR; and follow-up) as independent variables and the 6 previously listed sway parameters or the SSQ scores as dependent variables (in this case, the analysis was performed at sub scores and total score level). The level of significance was set at *p* = 0.05. When relevant, post hoc Holm–Sidak tests for pairwise comparison were used to assess intra- and inter-group differences. All the analyses were performed using IBM SPSS Statistics v.20 software (IBM, Armonk, NY, USA).

## 3. Results

### 3.1. Postural Sway

The results of the postural sway analysis are reported in Table 3 and Table 4 for the eyes open and eyes closed conditions, respectively.

In the presence of visual input, the only relevant (and statistically significant) change observed immediately after the VR session was represented by an increase in sway area for the group of pwMS who were more disabled. Such an alteration was partly recovered after 10 min follow-up. Instead, no substantial impairments of postural control were detected in both groups of pwMS with low disability and unaffected individuals. In greater detail, the statistical analysis revealed the presence of a significant main effect of group for all of the investigated parameters (*p* < 0.001), while a significant main effect of time (*p* = 0.006), as well as a significant time x group interaction (*p* = 0.049), was detected only for the sway area.

**Figure 2:**
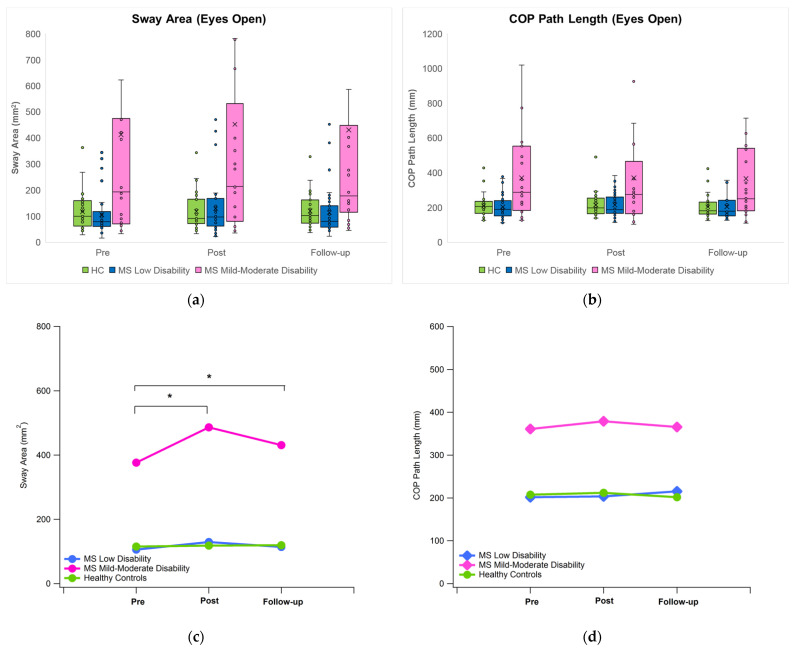
Trend of the sway area (left, panels (**a**,**c**)) and COP path length (right, panels (**b**,**d**)) for the eyes-open condition. Top: boxplots; bottom: mean values (the symbol * denotes the existence of a statistically significant difference for the “time” factor).

The trends of sway area and COP path length during the experimental tests are also provided in a graphical form in Figure 2. As previously mentioned, the post hoc analysis revealed that the only statistically significant effect associated with the exposure to immersive VR was represented by the increase in the sway area in pwMS with mild–moderate disability (+30% vs. pre-VR, *p* < 0.001) that persisted even after 10 min of rest (+14% vs. pre-VR condition, *p* = 0.05). As a matter of fact, a similar trend was also observed in pwMS with low disability (+22% vs. pre-VR condition), but in this case, statistical significance was not achieved. At last, unaffected individuals exhibit practically the same value for all three measurement points. In terms of group differences, as expected, the statistical analysis detected significant (higher) values in all sway parameters of pwMS with mild–moderate disability with respect to the remaining groups at each measurement timepoint. No differences were observed between unaffected individuals and pwMS with low disability.

**Figure 3:**
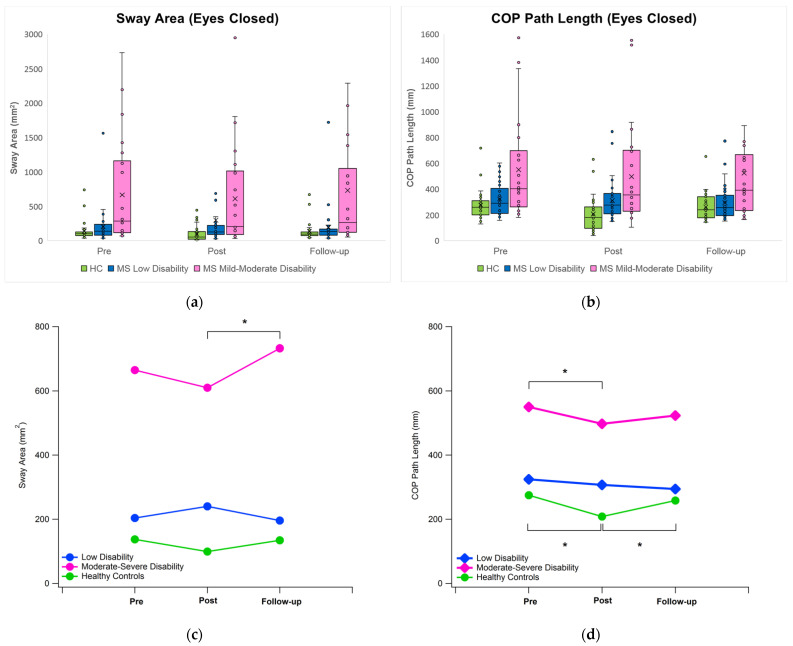
Trend of the sway area (left, panels (**a**,**c**)), and COP path length (right, panels (**b**,**d**)) for the eyes-closed condition. Top: boxplots; bottom: mean values (the symbol * denotes the existence of a statistically significant difference for the “time” factor).

In contrast, when the postural sway was measured in absence of visual input, the overall trend of variation during the experiment was completely different, particularly regarding s pwMS with mild–moderate disability and unaffected individuals (see Figure 3). In fact, statistical analysis detected a significant main effect of time only for the COP path length (*p* < 0.001) while (again) the main effect of the group existed for all the sway parameters, and significant time × group interactions were found in the case of sway area (*p* = 0.017), COP path length (*p* = 0.045), and COP velocity in ML direction (*p* = 0.001). In pwMS with mild–moderate disability, the sway area decreased after the exposure to VR, while the value at follow-up was found to be slightly higher with respect to the baseline (+10%) and significantly different with respect to the post-VR condition (*p* = 0.005). In the same group, a significant reduction in COP path length and COP velocity was observed after the VR session (10% vs. pre-VR in both cases; *p* = 0.015 for COP path length and *p* = 0.044 for COP velocity in ML). A similar trend was found for unaffected individuals, who were characterized by significantly reduced COP path length after the VR session (−24% vs. pre-VR, *p* < 0.001). However, at follow-up, the baseline value was restored.

Even in this case, group differences (similar to those previously described in the case of the eyes-open condition) were detected as they involved only pwMS with mild–moderate disability who exhibited sway parameters systematically (and significantly) larger than those of both unaffected individuals and pwMS with low disability.

### 3.2. Sickness Simulator Sickness Questionnaire

The total SSQ score, as well as the specific scores calculated for nausea, disorientation, and oculomotor domains, are reported in Table 5. The main effects of time were found via statistical analysis for all SSQ sub-scores (nausea, *p* = 0.090; oculomotor, *p* = 0.002; disorientation, *p* < 0.001) and for the total score (*p* < 0.001). A main effect in terms of group was also detected, but in this case, it only regarded oculomotor disturbance and total score. The post hoc analysis revealed that all scores (except those of the oculomotor domain for the group of pwMS with mild–moderate disability) increase significantly after exposure to the immersive VR. The largest changes involved the disorientation domain, with particular emphasis on symptoms like blurred vision, dizziness (both in eyes open and eyes closed conditions) and nausea. Instead, group effects were restricted to pwMS with mild–moderate disability who exhibited significantly higher pre-VR scores in the oculomotor domain when compared with unaffected individuals (*p* = 0.015) and with pwMS with low disability (*p* = 0.010) and significantly higher post-VR scores with respect to pwMS with low disability (*p* = 0.047).

## 4. Discussion

### 4.1. General Remarks and Effect of Immersive VR on Postural Sway under Eyes Open Condition

The main purpose of the present study was to characterize the magnitude and features of possible adverse effects (i.e., cybersickness) experienced by pwMS with different levels of disability who underwent a short session of highly stimulating immersive VR. Considering that previous studies demonstrated that cybersickness affects indistinctly both healthy individuals and those with neurologic conditions [3,14], a secondary aim of the study was to assess if (and to what extent) the specific features of MS exacerbate cybersickness and, among pwMS, clarify the role played by disability in terms of symptom severity. The analysis was carried out by integrating subjective perceptions of discomfort (quantified using the SSQ questionnaire) and objective instrumental measurements of postural control system effectiveness (i.e., postural sway).

At first, consistent with most existing studies, our results confirm that pwMS are characterized by postural control impairments that tend to worsen as the disease progresses [31,32]. The level of disability also seems to play a relevant role in terms of changes in sway parameters related to VR exposure under the eyes open condition. In particular, pwMS with low disability react in a way similar to unaffected individuals, as most of their sway parameters were found to be practically unaltered in terms of the post-VR and follow-up measurements and substantially coincident, thus suggesting a 10 min exposure to even highly stimulating immersive VR is not capable of triggering noticeable alterations in postural control. In contrast, pwMS with mild–moderate disability seemed more susceptible to the simulation, as demonstrated by the relevant increase observed in sway area and COP maximum displacements values and considering that a residual (yet significant) alteration of sway area persists at follow-up.

Generally speaking, alterations in postural control subsequent to immersive VR use were previously observed in healthy individuals [23,33] and hypothesized to be related to sensory conflict. In particular, the afferent pathways involved in postural control regulation receive contrasting information during VR sessions as the visual input is deceived by very realistic illusions of self-motion, while both the vestibular and somatosensory systems are not stimulated at all, thus communicating to the central nervous system (CNS) that the body is actually motionless. This leads to sensory mismatch that the CNS tries to cope with through the use of suitable adaptation strategies [34]. However, as VR exposure ceases and the individual returns to the real physical environment, such adaptations become counterproductive and negatively affect postural control effectiveness. In the present study, such an effect was observed only regarding the sway area of pwMS with mild–moderate disability. A possible explanation for this phenomenon lies in the fact that, as the disease progresses, postural control impairments become more severe, as indicated by the increase in sway parameters [32,35]. Thus, it is possible that the reduced effectiveness of the postural control system increases the individual’s susceptibility to the sensory conflict triggered by VR exposure. On the other hand, in both unaffected individuals and pwMS with low disability, no postural sway alterations were observed, probably due to the limited period of exposure, which, in this case, might not be sufficient to negatively impact postural control system performance. It is noteworthy, though, that the literature is quite controversial regarding the time required to provoke the onset of cybersickness since several studies have reported values shorter than 10 min, while others found that larger amounts of time are necessary and, moreover, longer exposure durations were found to be significantly associated with more severe symptoms [16].

Another interesting aspect that emerged from the analysis of the sway data in the presence of visual input is represented by the lack of impact of VR on COP path length and COP velocities in all groups, contrary to what has been reported in previous studies that detected significant post-VR increases [33]. Such an apparent contradiction can be explained by the different postures assumed by the participants (sitting in our study; standing in [33]). In fact, it should be recalled that, according to the most accepted interpretation, COP path length and COP velocity provide information on the corrections required to maintain postural stability (i.e., longer paths are likely to reflect a higher number of postural corrections), and thus they can be considered indicators of the muscular effort produced to control body oscillations [36]. Thus, it is likely that in participants exposed to VR in a sitting position, the muscles involved in the control of the upright posture (particularly those of the ankle joint) were scarcely or not at all engaged, which is different from what is likely to occur when individuals assume a standing position. This latter condition certainly induces a relevant muscular activity that is able to increase COP path length and velocity to simultaneously cope with gravity and sensory conflict induced by VR.

### 4.2. Effect of Immersive VR on Postural Sway under Eyes Closed Condition

Quite surprisingly, the results drastically changed when postural sway acquired in the absence of visual input was analyzed as, in this case, the exposure to immersive VR was associated with a general trend of improvement in sway parameters. This suggests that, especially in pwMS with mild–moderate disability, VR might exert a sort of “training” effect, which is only present when the postural control system is deprived of one of the afferent pathways. Although a direct comparison of such findings with previous data is difficult due to the scarcity of similar studies on pwMS, it is noteworthy that in several previous studies carried out on healthy young adults [37,38], postural control effectiveness was found to be similar under eyes closed condition (no VR) and during a VR session (that is while wearing the HMD) in which either static or highly stimulating media were delivered. Such phenomenon was explained by hypothesizing that being exposed to a VR environment using a HMD is equivalent to a condition in which the visual input is removed since the weight and quality of the information provided by the visual system to the CNS are somehow altered. Based on this assumption, exposure to VR would act as a sort of “balance training” and, as a result, immediately after VR exposure, the postural control of the individual, which previously underwent some adaptation to reweight and better exploit the remaining proprioceptive and vestibular information, would perform better.

### 4.3. Effect of Immersive VR on Subjective Perception of Cybersickness

As expected, the baseline scores of the SSQ revealed that pwMS with mild–moderate disability approached the VR session with the burden of a symptomatology that partly overlaps with those assessed using the questionnaire. It was also observed that, after the immersive VR session, both healthy individuals and pwMS with low disability experienced significant increases in cybersickness symptoms, which involved all three domains and the total score. Although no statistically significant group differences were detected, it is noticeable that unaffected individuals reported even larger pre-VR vs. post-VR changes with respect to pwMS with low disability. The mean total score of 26.5 calculated for unaffected individuals is consistent with values obtained in similar previous experiences, but it should also be noted that in our sample, we observed quite large variability (SD 32.5). This is probably due to the different susceptibility to cybersickness associated with the age of the participants. In fact, in order to have groups suitably matched, the age of unaffected individuals ranged from 26 to 64 years old and, thus, it is possible that older participants experienced different levels of sickness than younger (as previously observed by Kennedy et al. [39] and Keshavarz et al. [40]), making the results less homogeneous.

The highest post-VR SSQ scores were obtained by pwMS with mild–moderate disability, but the differences with the other groups were significant only concerning the oculomotor domain in comparison with pwMS with low disability (22.05 vs. 9.26, *p* = 0.047). It is also noteworthy that, in those with greater disability, pre-VR vs. post-VR increases in all scores were the lowest among the three tested groups. Such results suggest that the assessment of subjective discomfort within a rehabilitative/training program that makes use of immersive VR must be carried out by acquiring data prior to the session. This is crucial in order to correctly estimate the actual impact of the treatment, considering the existing magnitude of the symptoms attributable to MS but also present in the SSQ. At last, although, as previously mentioned, only a few similar studies performed on a small sample of pwMS exist, and the total score values post-VR calculated in this study (which ranges from 17.87 to 31.89 depending on the disability level) are consistent with those reported by Arafat et al. [18,19] for pwMS, having EDSS from 4 to 4.5 (mean 33.1, SD 31.7).

### 4.4. Limitations of the Study

Some limitations of the study should be acknowledged. Firstly, we employed a single technique, namely postural sway analysis, to objectively assess cybersickness. Although this represents the most employed approach to this purpose, changes in other physiological measurements (i.e., electroencephalogram, electrogastrogram, electrocardiogram and electrodermal activity) might provide complementary valuable information [16]. Secondly, it is recognized that cybersickness is influenced by factors like duration of the exposure, hardware and software features, gender, age, familiarity with the use of videogames/VR and habit to repeated exposures, etc., which have not been investigated in detail here and should be considered in further studies on pwMS. Moreover, in the present study, participants were required to use VR in a sitting position due to the wide spectrum of variability, which would have made it unsafe to use in the standing position for some of them. However, since immersive VR has been successfully employed to train balance [41], where possible, the standing position should also be adopted for pwMS who can safely maintain it during the entire VR session. Thus, additional investigations on how cybersickness features change using a variety of postures should be carried out. At last, the rollercoaster simulation employed here is probably among those that most easily trigger severe cybersickness symptoms, given the extreme realism in terms of motion sensation. It is likely that the software employed for rehabilitation/training of pwMS has relatively more static contents and, as such, is less prone to produce unpleasant effects. However, the dosage to immersive VR in terms of exposure time should always be tailored to each pwMS considering not only its specific reactivity (possibly assessed through a combination of subjective and objective measures) but also the existence of possible effects of desensitization (i.e., reduction in cybersickness due to repeated exposure to the same content) which might attenuate adverse effects with time [42].

## 5. Conclusions

Although immersive VR can be considered a promising technology capable of supporting the rehabilitation and training of pwMS, its use should be carefully planned considering the occurrence of possible unpleasant side effects not only regarding those subjectively perceived but also in terms of objectively assessing impairments in postural control, which is potentially hazardous as it is associated with an increased risk of falls. Our study demonstrated that even short exposure to strongly stimulating VR scenarios can have significant effects on postural control of pwMS depending on their disability level and on the availability of the visual input. Additionally, most pwMS reported moderate to severe cybersickness symptoms after the VR exposure, but substantially, the effect was similar to what was observed in healthy individuals, even though it should be recalled that they were likely to approach the VR session with symptoms of the same type of those analyzed in the SSQ. This may indicate the inadequacy of such a questionnaire for pwMS and, thus, suggest the development and use of dedicated tools to accurately assess the actual impact of an immersive VR session. In short, although the type and magnitude of possible side effects associated with immersive VR exposure seem extremely variable from individual to individual, its use seems sufficiently safe and tolerable to not be contraindicated in the rehabilitation/training of pwMS. However, considering that the use of immersive VR is becoming widespread in rehabilitation centers specialized in MS, it appears necessary to develop and adopt specific guidelines to minimize the possible adverse effects and to maximize efficacy, safety and comfort for pwMS. Such document should, at the very least, include recommendations on the following: an initial assessment of individual susceptibility to cybersickness (for instance, using dedicated questionnaires), maximum continuative exposure time (based on individual susceptibility and the level of optical flow of the simulation), type of posture to adopt during VR training (i.e., standing or sitting depending on the disability level and on the need to challenge balance) and overall assessment of cybersickness after the VR exposure both in terms of perceived discomfort as well as with objective experimental techniques.

## Figures and Tables

**Table 1:** List of symptoms included in the Simulator Sickness Questionnaire [17]. The symbol X indicates the inclusion of a certain symptom in the calculation of score for a specific domain.

	Domain		
Symptom	*Nausea*	*Oculomotor* *Disturbance*	*Disorientation*
General Discomfort	X	X	
Fatigue		X	
Headache		X	
Eyestrain		X	
Difficulty Focusing		X	X
Increased Salivation	X		
Sweating	X		
Nausea	X		X
Difficulty Concentrating	X	X	
Fullness of Head			X
Blurred Vision		X	X
Dizzy (eyes open)			X
Dizzy (eyes closed)			X
Vertigo			X
Stomach Awareness	X		
Burping	X		

**Table 2:** Anthropometric features of the tested participants. Values are expressed as mean (SD).

	Healthy Controls	All MS	MS Low Disability (EDSS 0–2.0)	MS Mild–Moderate Disability (EDSS 2.5–6.5)
**Participants**	33 (29 F, 4 M)	56 (46 F, 10 M)	34 (28 F, 6 M)	22 (18 F, 4 M)
**Age (years)**	47.6 (10.5)	45.3 (13.1)	41.8 (13.4) *	50.7 (10.7)
**Height (cm)**	161.7 (7.5)	163.0 (7.7)	164.2 (7.1)	161.2 (7.1)
**Body Mass (kg)**	63.3 (12.9)	62.9 (12.6)	64.7 (12.5)	60.1 (12.5)
**EDSS score**	-	2.3 (1.4)	1.3 (0.6)	3.7 (1.1)
**Type of MS**	-	44 RR/4 PP/8 SP	34 RR	10 RR/4 PP/8 SP
**Disease duration (years)**	-	6.1 (5.8)	4.8 (6.2)	8.2 (6.4)

The symbol * denotes the existence of a statistically significant difference vs. healthy controls. EDSS: Expanded Disability Status Scale; M: male; F: female; MS: multiple sclerosis; RR: relapsing–remitting; PP: primary progressive; SP: secondary progressive.

**Table 3:** Postural sway parameters under eyes-open conditions before and after exposure to VR and at 10 min follow-up. Values are expressed as mean (SD).

Group	Time	Sway Area	COP Path Length	COP Disp. ML	COP Disp. AP	COP Velocity ML	COP Velocity AP
*HC*	Pre	115.49 (69.17)	207.64 (62.88)	12.88 (5.71)	17.83 (5.00)	4.33 (1.30)	4.76 (1.45)
	Post	118.05 (67.17)	212.04 (68.13)	12.45 (4.40)	19.88 (6.19)	4.32 (1.48)	4.95 (1.57)
	FU	119.47 (63.57)	201.86 (63.19)	13.17 (5.77)	19.15 (5.50)	4.16 (1.39)	4.66 (1.37)
*MS Low*	Pre	105.87 (79.03)	203.61 (68.44)	12.17 (5.66)	16.93 (6.15)	4.25 (1.44)	4.70 (1.68)
	Post	129.29 (105.28)	215.40 (71.84)	12.99 (6.12)	19.16 (6.51)	4.45 (1.42)	5.01 (1.84)
	FU	114.03 (93.56)	204.07 (65.48)	12.29 (5.75)	19.03 (7.28)	4.22 (1.32)	4.75 (1.62)
*MS Mod*	Pre	376.24 (479.08) †‡	361.06 (244.10) †‡	23.90 (19.84) †‡	25.77 (13.93) †‡	7.66 (5.63) †‡	8.05 (5.09) †‡
	Post	486.40 (674.65) *†‡	379.08 (318.98) †‡	25.99 (21.39) †‡	30.62 (20.05) †‡	8.04 (7.19) †‡	8.47 (6.76) †‡
	FU	430.94 (587.54) *†‡	365.76 (297.36) †‡	23.54 (17.38) †‡	29.63 (17.19) †‡	7.81 (7.02) †‡	8.12 (6.10) †‡

MS: multiple sclerosis; COP: center of pressure; Disp.: displacements; FU: follow-up; ML: medio–lateral; AP: antero–posterior. The symbol * denotes the existence of a statistically significant difference vs. pre-VR condition. The symbol † denotes the existence of a statistically significant difference vs. healthy controls. The symbol ‡ denotes the existence of a statistically significant difference vs. MS low disability.

**Table 4:** Postural sway parameters under eyes-closed conditions before and after exposure to VR and at 10 min follow-up. Values are expressed as mean (SD).

Group	Time	Sway Area	COP Path Length	COP Disp. ML	COP Disp. AP	COP Velocity ML	COP Velocity AP
*HC*	Pre	137.09 (135.84)	274.98 (110.39)	14.37 (7.55)	20.02 (6.35)	5.15 (1.84)	6.81 (3.00)
	Post	99.09 (107.12)	208.09 (131.16) *	13.73 (7.32)	17.42 (8.26)	6.78 (2.83) *	5.88 (2.66)
	FU	134.12 (131.17)	258.19 (104.52) **	14.00 (7.60)	19.78 (8.23)	4.90 (1.95) **	6.33 (2.66)
*MS Low*	Pre	203.64 (262.64)	324.41 (131.17)	15.58 (7.88)	25.01 (9.53)	6.11 (2.61)	8.05 (3.25)
	Post	240.24 (425.12)	306.84 (138.88)	16.88 (11.74)	25.49 (12.67)	5.68 (2.67)	7.70 (3.43)
	FU	195.97 (291.44)	294.04 (135.57)	14.25 (9.90)	24.13 (10.26)	5.47 (2.65)	7.36 (3.35)
*MS Mod*	Pre	664.95 (777.82) †‡	549.94 (408.54) †‡	29.63 (20.40) †‡	36.30 (18.95) †‡	10.62 (7.88) †‡	13.23 (9.94) ‡
	Post	609.80 (754.38) †‡	497.37 (405.85) *†‡	26.29 (20.42) †‡	37.82 (22–18) ‡	9.47 (7.77) *‡	12.11 (9.95) ‡
	FU	732.52 (954.22) **†‡	523.22 (422.10) †‡	29.86 (24.42) †‡	38.37 (21.70) ‡	10.34 (8.96) †‡	12.29 (9.24) ‡

MS: multiple sclerosis; COP: center of pressure; Disp.: displacements; FUp: follow-up; ML: medio–lateral; AP: antero–posterior. The symbol * denotes the existence of a statistically significant difference vs. Pre-VR condition. The symbol ** denotes the existence of a statistically significant difference vs. Post-VR condition. The symbol † denotes the existence of a statistically significant difference vs. healthy controls. The symbol ‡ denotes the existence of a statistically significant difference vs. MS low disability.

**Table 5:** SSQ scores before and after exposure to VR. Values are expressed as mean (SD).

	Healthy Controls		MS Low Disability EDSS 0–2.0		MS Mild–Moderate Disability EDSS 2.5–6.5	
	Pre-VR	Post-VR	Pre-VR	Post-VR	Pre-VR	Post-VR
**Nausea**	0.00 (0.00)	28.35 (33.17) *	1.06 (3.04)	14.31 (21.21) *	12.14 (24.13)) ^†‡^	22.98 (33.78)
**Oculomotor**	0.00 (0.00)	11.48 (18.14) *	3.58 (7.79)	9.26 (12.83) *	18.95 (32.64) ^†‡^	22.05 (29.21) ^‡^
**Disorientation**	0.80 (3.28)	36.19 (46.07) *	3.48 (10.72)	28.61 (41.55) *	24.68 (39.59)	44.29 (53.07) *
**Total Score**	0.21 (0.88)	26.50 (32.50) *	3.12 (7.12)	17.87 (23.94) *	20.74 (34.81)	31.79 (36.89) *

The symbol * denotes the existence of a statistically significant difference vs. pre-VR condition. The symbol † denotes the existence of a statistically significant difference vs. healthy controls. The symbol ‡ denotes the existence of a statistically significant difference vs. MS low disability.

## Data Availability

Data will be made available upon request.
